# Circulating microRNAs are associated with early childhood obesity: results of the I.Family Study

**DOI:** 10.1186/s12263-018-0622-6

**Published:** 2019-01-09

**Authors:** Giuseppe Iacomino, Paola Russo, Pasquale Marena, Fabio Lauria, Antonella Venezia, Wolfgang Ahrens, Stefaan De Henauw, Pasquale De Luca, Ronja Foraita, Kathrin Günther, Lauren Lissner, Dénes Molnár, Luis A. Moreno, Michael Tornaritis, Toomas Veidebaum, Alfonso Siani

**Affiliations:** 1 Institute of Food Sciences, National Research Council, ISA-CNR, Via Roma, 64 83100 Avellino, Italy; 2 Leibniz-Institute for Prevention Research and Epidemiology, BIPS, Bremen, Germany; 3 University of Ghent, Ghent, Belgium; 4 Stazione Zoologica Anton Dohrn, Naples, Italy; 5 Sahlgrenska Academy at the University of Gothenburg, Gothenburg, Sweden; 6 Medical School, University of Pécs, Pécs, Hungary; 7 University of Zaragoza, Zaragoza, Spain; 8 Research and Education Institute of Child Health, Strovolos, Cyprus; 9 National Institute for Health Development, Tallinn, Estonia

**Keywords:** Circulating miRNAs, Childhood obesity, Childhood overweight/low-grade obesity, Metabolic disorders, Biomarker

## Abstract

**Background:**

Nearly 10 years ago, the World Health Organization reported the increasing prevalence of overweight and obesity worldwide as a challenge for public health due to the associated adverse consequences. Epidemiological studies established a firm relationship between an elevated body mass index and chronic conditions such as diabetes, dyslipidemia, hypertension, heart disease, non-alcoholic fatty liver disease, and some types of cancer. Omic studies demonstrated that microRNA (miRNA) profile changes in tissues correlate with a number of diseases, including obesity. Recent studies showed a remarkable stability of miRNAs also in blood, emphasizing their potential as theranostic agents for a variety of disorders and conditions. A number of miRNAs enriched in homeostasis of obesity and metabolic disorders have been characterized in previous researches.

**Aim:**

This work was finalized to investigate the differential circulating miRNAs signature in early childhood obesity. Our cross-sectional study analyzed the signature of circulating miRNAs in plasma samples of normal weight (*n* = 159) and overweight/obese (*n* = 149) children and adolescents participating to the I.Family study, an EC-funded study finalized to investigate the etiology of overweight, obesity and related disorders and the determinants of food choice, lifestyle, and related health outcomes in children and adolescents of eight European countries (www.ifamilystudy.eu).

**Results:**

Differences in miRNA signature with respect to anthropometric and biochemical variables were analyzed. A high degree of variability in levels of circulating miRNAs was identified among children from different countries, in line with recent reports supporting the hypothesis that these molecules are likewise affected by environmental and lifestyle factors. A panel of miRNAs differentially expressed in overweight/low-grade obesity children was characterized (miR-551a and miR-501-5p resulted upregulated; miR-10b-5p, miR-191-3p, miR-215-5p, and miR-874-3p resulted downregulated). ROC curves were also constructed for experimentally confirmed miRNAs. Single miRNAs generally exhibited low AUC values with the highest values for miR-874-3p and miR-501-5p which in combination provided an interesting value (AUC = 0.782). Pearson’s analysis confirmed that miR-10b-5p, miR-215-5p, miR-501-5p, miR-551a, and miR-874-3p significantly correlated with BMI *z*-score. Molecular interactions of obesity-associated miRNAs were also predicted by bioinformatics tools.

**Conclusions:**

Our work showed that several circulating miRNAs are differentially represented in overweight/low-grade obesity children and adolescents. Although causal pathways cannot be firmly inferred, it is conceivable that circulating miRNAs may be new biomarkers of early childhood obesity.

**Trial registration:**

ISRCTN, ISRCTN62310987. Registered 23/02/2018 - Retrospectively registered.

**Electronic supplementary material:**

The online version of this article (10.1186/s12263-018-0622-6) contains supplementary material, which is available to authorized users.

## Background

In recent times, the discovery of microRNAs (miRNAs) has contributed to the wide range of epigenetic mechanisms related to obesity. In 2016, a pilot study was conducted by our group on a sample of overweight/obese (OW/Ob) children belonging to the Italian cohort of the I.Family project [1], an EC-funded study finalized to investigate the etiology of overweight, obesity and related disorders and the determinants of food choice, lifestyle, and related health outcomes in children and adolescents of eight European countries [2].

miRNAs are short non-coding RNAs with a length of 20–24 nucleotides, which are involved in the fine control of the gene expression [3]. At present, more than 2500 different miRNAs have been described in humans. Release 22 (March 2018) of miRBase database contains 38,589 entries representing hairpin precursor miRNAs, expressing 48,885 mature miRNA products, in 271 species (http://www.mirbase.org/). Post-transcriptional gene regulation is achieved by miRNAs through mechanisms of translational inhibition and mRNA destabilization. Remarkably, each miRNA can target many transcripts, and individual mRNA may include multiple binding sites for different miRNAs. The concurrent targeting of multiple genes can lead to a specific fine-tuning through the regulation of distinct sub-networks [4].

A number of miRNAs have been confirmed to be present in surprisingly high concentrations in serum/plasma and other body fluids [5]. The evidence that miRNAs could be stable in blood, in spite of the ubiquity of nucleases, was originally met with skepticism by scientists; however, this finding generated high interest for the possibility that changes in cell-free miRNAs could be used as stable and accessible indicators for a variety of physio-pathological conditions [6]. Although a recognized mechanism for the release of miRNAs from cells is mainly indefinite, growing evidence supports the indication that extracellular miRNAs, arranged either into exosomes or protein complexes, may be delivered to the receiver cells, where they can be involved in the control of target gene translation. Nevertheless, the physiological role of circulating miRNAs remains still uncertain.

Considerable progress has been achieved in the research of contributory crosstalk between miRNAs and metabolic disorders, and recently, a number of miRNAs have been recognized to be involved in adipogenesis, adipose tissue metabolism, and obesity [1, 7–9]. Inclusively, a number of miRNAs enriched in homeostasis of obesity and metabolic disorders have been earlier reviewed [10]. Moreover, various studies have revealed a differential circulating miRNAs signature in overweight/obese as compared to normal weight children and adolescents.

The aim of this investigation was to identify, in a larger sample of children belonging to the I.Family Project, circulating miRNAs potentially associated with primary stages of obesity via an integrated study comprising miRNA signatures and bioinformatic analyses in order to shed light on miRNA regulatory networks in early childhood obesity. This is a validation study seeking for confirmation of previous results and need to be envisaged as such.

## Results

### Anthropometric and metabolic characteristics of the study population

Individual plasma samples (*n* = 308, NW = 159, OW/Ob = 149) were first screened for hemolysis, and hemolyzed samples were excluded from analysis. The anthropometric and metabolic characteristics of the 189 resulting participants are summarized in Tables 1 and 2 respectively. The average BMI *z*-score in the OW/Ob group was 1.75 ± 0.61, compared to − 0.04 ± 0.50 in the NW group. OW/Ob children also had significantly higher triglyceride levels, insulin levels, HOMA index, and lower HDL levels as compared to NW; total cholesterol and LDL levels were not significantly different between the two groups.

**Table 1:** Characteristics of subjects included in the study

	NW			OW/Ob		
	*N* (M/F)	Age (years)	BMI *z*-score	*N* (M/F)	Age (years)	BMI *z*-score
Belgium	15 (6/9)	11.6 ± 1.5	− 0.26 ± 0.04	7 (3/4)	11.8 ± 2.1	1.44 ± 0.42
Cyprus	6 (2/4)	11.3 ± 1.6	− 0.15 ± 0.63	6 (2/4)	11.9 ± 1.3	1.96 ± 0.68
Estonia	17 (9/8)	13.0 ± 1.5	− 0.03 ± 0.48	14 (8/6)	13.2 ± 1.5	1.67 ± 0.48
Germany	10 (2/8)	12.1 ± 1.5	− 0.12 ± 0.56	16 (6/10)	13 ± 1.5	1.74 ± .60
Hungary	13 (8/5)	11.9 ± 2.0	0.06 ± 0.54	12 (4/8)	12.0 ± 2.3	1.96 ± 0.62
Italy	10 (2/8)	12.1 ± 1.3	0.18 ± 0.40	12 (9/3)	12.2 ± 1.4	1.97 ± 0.77
Spain	13 (9/4)	12.0 ± 2.3	0.08 ± 0.49	14 (4/10)	11.8 ± 1.9	1.66 ± 0.71
Sweden	11 (7/4)	11.4 ± 1.4	− 0.09 ± 0.54	13 (5/8)	11.2 ± 2.1	1.65 ± 0.47
All	95 (45/50)	12.0 ± 1.6	− 0.04 ± 0.50	94 (41/53)	12.3 ± 1.8	1.75 ± 0.61

*Nw* normal weight, *OW/Ob* overweight/obese. BMI *z*-score: age and sex-corrected body mass index. Values are expressed as mean ± SD. Subjects from the distinct countries correspond to separate pools of NW and OW/Ob

**Table 2:** Metabolic characteristics of subjects included in the study

		NW	OW/Ob	*p*
Glucose	(mg/dl)	92.8 ± 6.7	94.0 ± 7.4	0.257
Insulin	(pg/ml)	238.1 ± 182.1	385.6 ± 323.8	< *0.001*
Homa index		1.4 ± 1.0	2.2 ± 1.9	*0.001*
HBA1	(%)	5.0 ± 0.3	4.9 ± 0.3	0.494
Triglycerides	(mg/dl)	63.2 ± 28.8	78.5 ± 45.0	*0.006*
Total cholesterol	(mg/dl)	157.6 ± 24.9	153.2 ± 22.7	0.212
HDL cholesterol	(mg/dl)	62.0 ± 14.1	52.8 ± 11.2	*< 0.001*
LDL cholesterol	(mg/dl)	86.9 ± 22.0	88.9 ± 20.7	0.522

*Nw* normal weight, *OW/Ob* overweight/obese, *LDL* low-density lipoprotein, *HDL* high-density lipoprotein, *HOMA index* homeostasis model assessment of insulin resistance, *HBA1* hemoglobin A1c. Data are expressed as mean ± SD

### qPCR-arrays screening and RT-qPCR validation of candidate miRNAs

**Fig. 1:**
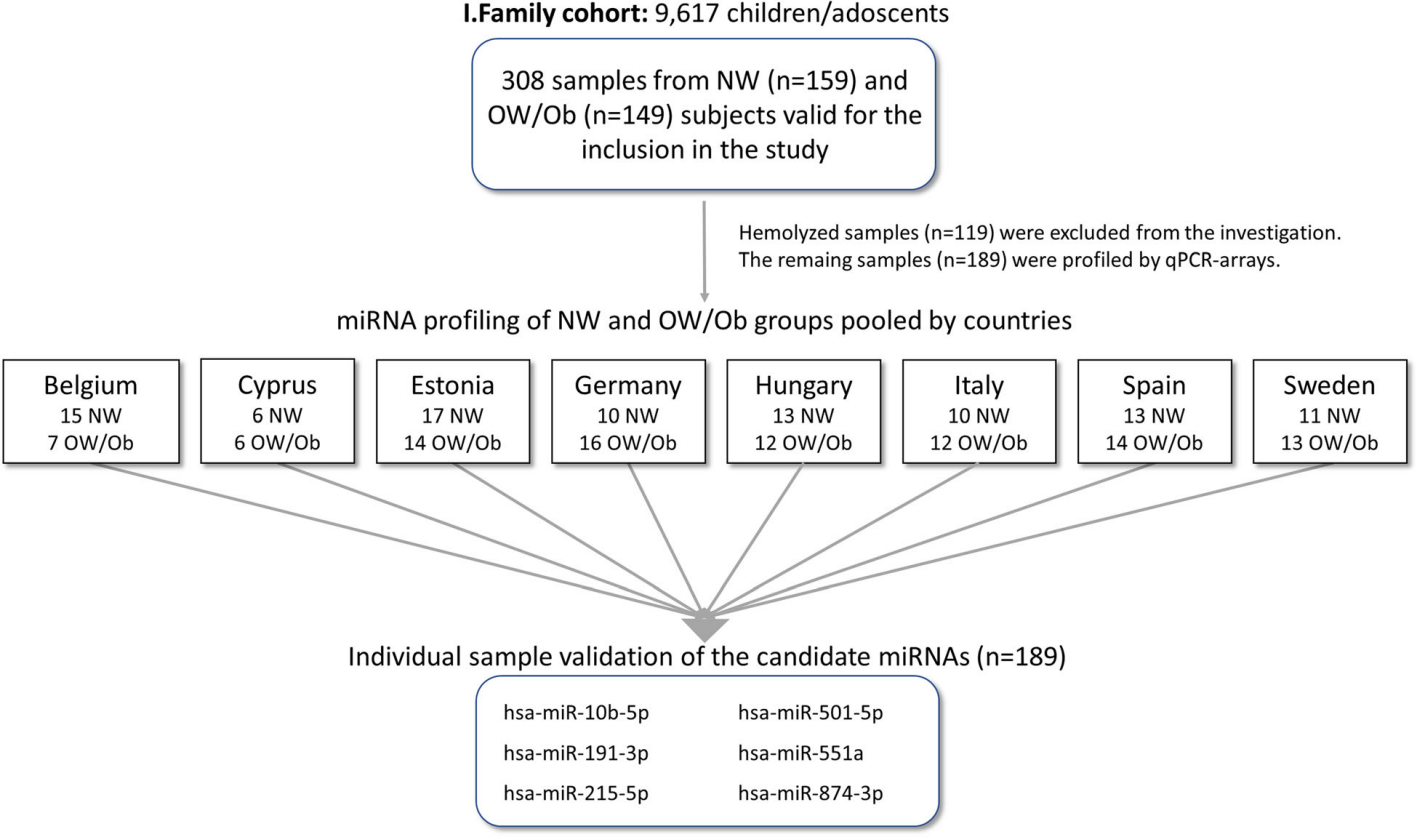
Schematic flow diagram of the proposed approach for the identification of miRNA patterns in early obesity

**Fig. 2:**
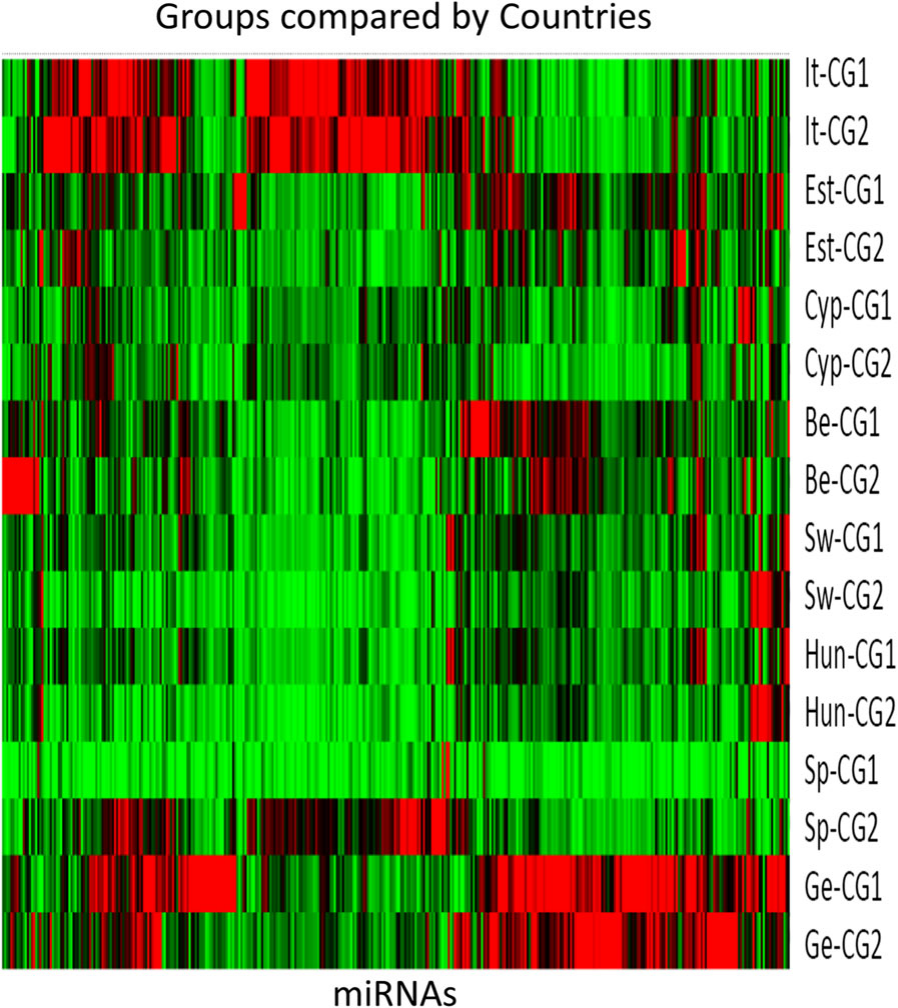
Hierarchical clustering analysis. Differences were appreciable between the compared groups but a high degree of variability was also recognized for the different countries

As a time and cost-reducing strategy, samples from NW and OW/Ob subjects were preliminarily extracted and evaluated as pools grouped by country of origin (Fig. 1). These groups were investigated for diverse countries by PCR arrays performed in triplicate. Subsequent data processing included the scatter plot analysis, useful to identify changes in magnitude and relative abundance of single circulating miRNAs in OW/Ob vs NW group. The statistical significance of miRNA levels between NW and OW/Ob groups was evaluated by the volcano plot analysis combining the transformed *p* value as a measure of statistical significance with the magnitude of the fold change (Additional file 1: Figure S1). The significance level for selected miRNAs was in general low. Based on the statistical significance (*p* < 0.05), 9 miRNAs were preliminarily selected as candidates for the RT-qPCR validation step. Moreover, 5 extra miRNAs selected among the best of those discarded (*p* > 0.05, and > 1.7-fold change difference between groups) were also included in the confirmatory study (Table 3). miRNAs co-regulated patterns were also investigated by hierarchical clustering to characterize miRNA signature by countries (Fig. 2).

**Table 3:** Selected candidate miRNAs

miRNA	miRBase accession number
hsa-miR-10b-5p	MIMAT0000254
hsa-miR-26b-3p	MIMAT0004500
hsa-miR-31-5p	MIMAT0000089
hsa-miR-191-3p	MIMAT0001618
hsa-miR-206	MIMAT0000462
hsa-miR-215-5p	MIMAT0000272
hsa-miR-375	MIMAT0000728
hsa-miR-483-5p	MIMAT0004761
hsa-miR-485-5p	MIMAT0002175
hsa-miR-501-5p	MIMAT0002872
hsa-miR-551a	MIMAT0003214
hsa-miR-576-5p	MIMAT0003241
hsa-miR-874-3p	MIMAT0004911
hsa-miR-2355-5p	MIMAT0016895

Based on the fold change and/or statistical significance, 14 miRNAs were preliminarily selected as candidates miRNAs for RT-qPCR validation

### RT-qPCR validation in individual plasma samples

Designated miRNAs were further evaluated in individual samples by RT-qPCR using the whole cohort of 189 subjects. Following an extraction of single samples, miRNA relative levels were normalized using the spike-in Cel-miR-39. Data were next clustered into control (NW) and OW/Ob groups, and miRNA expression was normalized to the average of the NW group. In general, only slight differences in expression between compared groups were found. Nevertheless, when statistical analysis of miRNAs expression was performed by volcano plot analysis, using a *p* value cutoff of < 0.05 and Benjamini-Hochberg false discovery rate method for the multiple testing corrections, several miRNAs were identified (miR-10b-5p, *p* = 0.000009; miR-191-3p, *p* = 0.004; miR-215-5p, *p* = 0.0002; miR-551a, *p* = 0.005; miR-874-3p, *p* = 0.0001). Moreover, as reported in Table 4, the differential expression of a subset of candidate miRNAs was statistically confirmed by analysis of covariance (ANCOVA) adjusting for covariates (age, sex, and country of origin).

**Table 4:** Statistically significant deregulated miRNAs

	NW	OW/Ob	*p*	Adj *R*^2^
hsa-miR-10b-5p	3.885 (3.421–4.349)	3.096 (2.632–3.560)	0.019	0.037
hsa-miR-191-3p	4.255 (3.684–4.827)	3.381 (2.809–3.952)	0.035	0.033
hsa-miR-215-5p	2.925 (2.407–3.443)	2.134 (1.613–2.654)	0.035	0.028
hsa-miR-501-5p	0.589 (0.503–0.676)	0.799 (0.712–0.886)	0.001	0.126
hsa-miR-551a	0.102 (0.065–0.140)	0.173 (0.135–0.211)	0.010	0.033
hsa-miR-874-3p	5.633 (5.064–6.202)	3.935 (3.363–4.508)	< 0.001	0.067

*NW* normal weight, *OW/Ob* overweight/obese. Values are mean [95% confidence interval (CI)], adjusted for age, sex, and country of origin. Covariates effect: miR-10b-5p: country (*p* = 0.019); miR-191-3p: none; miR-215-5p: none; miR-501-5p: country (*p* < 0.001); miR-551a: none; miR-874-3p: none

### Correlation of circulating miRNAs to BMI *z*-score and biochemical parameters

**Fig. 3:**
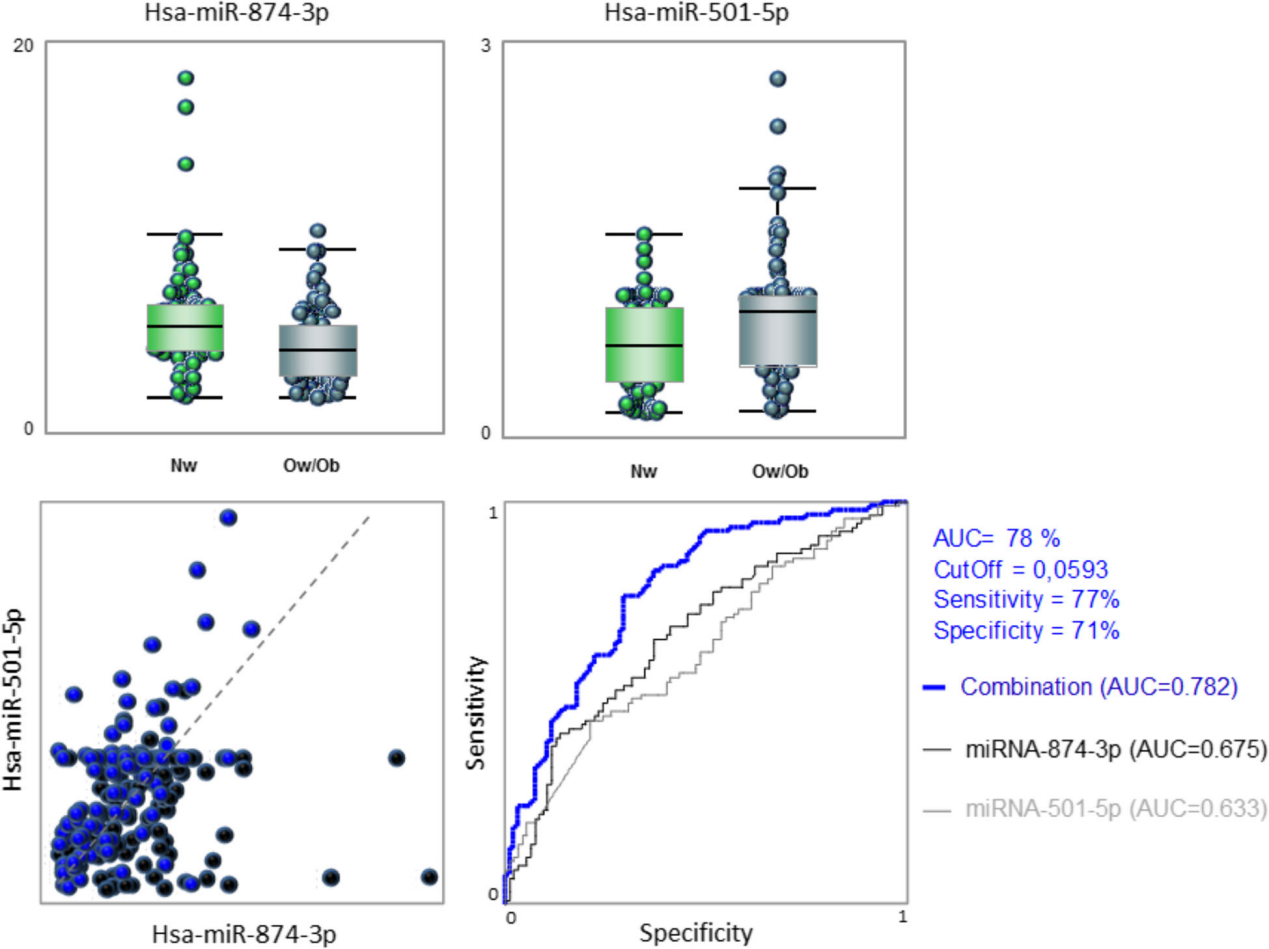
ROC curves to compare the ability of each miRNA to distinguish between groups. The AUC is a measure of how well a quantitative test can distinguish between OW/Ob and NW subjects. The area under the receiver–operator curve (AUC) for miR-874-3p and miR-501-5p are reported. Box-whisker charts are also reported in association with a scatter diagram for selected miRNAs

ROC curves were constructed for each validated miRNA and areas under the receiver–operator curve (AUC) were determined to evaluate the performance in discriminating between groups. The highest values were for miR-874-3p (AUC = 0.67) and miR-501-5p (AUC = 0.63) for which the respective ROCs are presented in Fig. 3 in association to box-whisker charts reporting the miRNA relative levels. When miR-874-3p and miR-501-5p were used in combination, this provided an interesting AUC = 0.782.

**Fig. 4:**
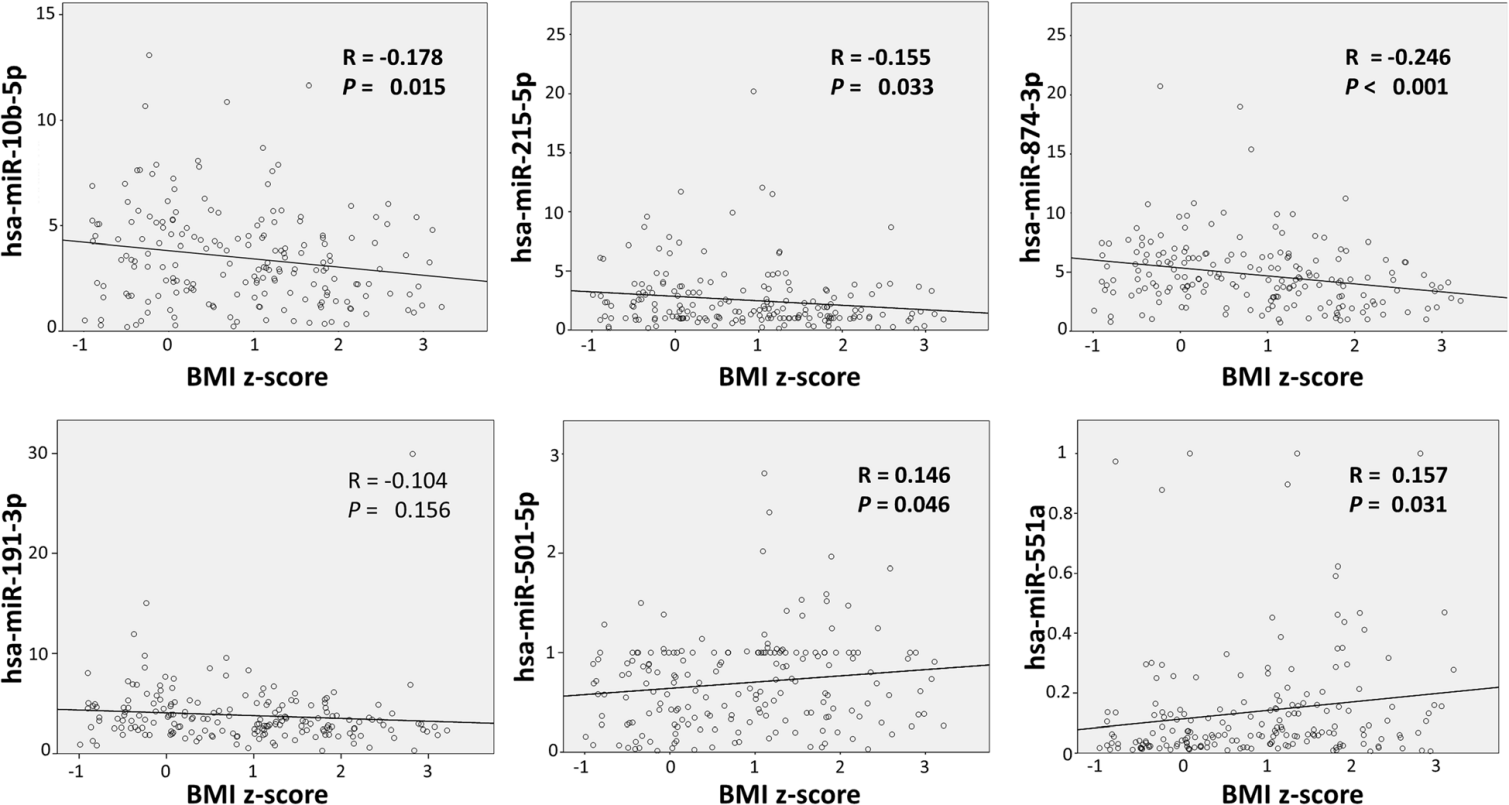
Correlation between miRNAs and the BMI *z*-scores. The correlation coefficient between the miRNA expression levels and BMI *z*-scores. *R* and *P* values are presented from Pearson’s analysis

Conceivable correlations among confirmed miRNAs levels and anthropometric/biochemical parameters were additionally evaluated by Pearson’s analysis. Correlation coefficients with BMI *z*-score were generally low. Nevertheless, for miR-10b-5p, miR-215-5p, miR-501-5p, miR-551a, and miR-874-3p associations with BMI, *z*-scores were confirmed as reported in Fig. 4 (in bold). MiR-874-3p, in addition to miR-501-5p, also correlated with the weight *z*-score (*R* = − 0.197, *p* = 0.007 and *R* = 0.172, *p* = 0.018, respectively). Associations with the biochemical parameters were also tested. None of the selected miRNAs correlated with levels of glucose, glycated hemoglobin, total cholesterol, HDL-C, and TRG (data not shown). Moreover, miR-191-3p, among miRNAs recognized in ANCOVA analysis, was associated with plasma insulin levels (*R* = 0.297, *p* < 0.01), and HOMA index (*R* = 0.313, *p* < 0.01) in Pearson’s analysis.

### miRNAs target prediction

**Fig. 5:**
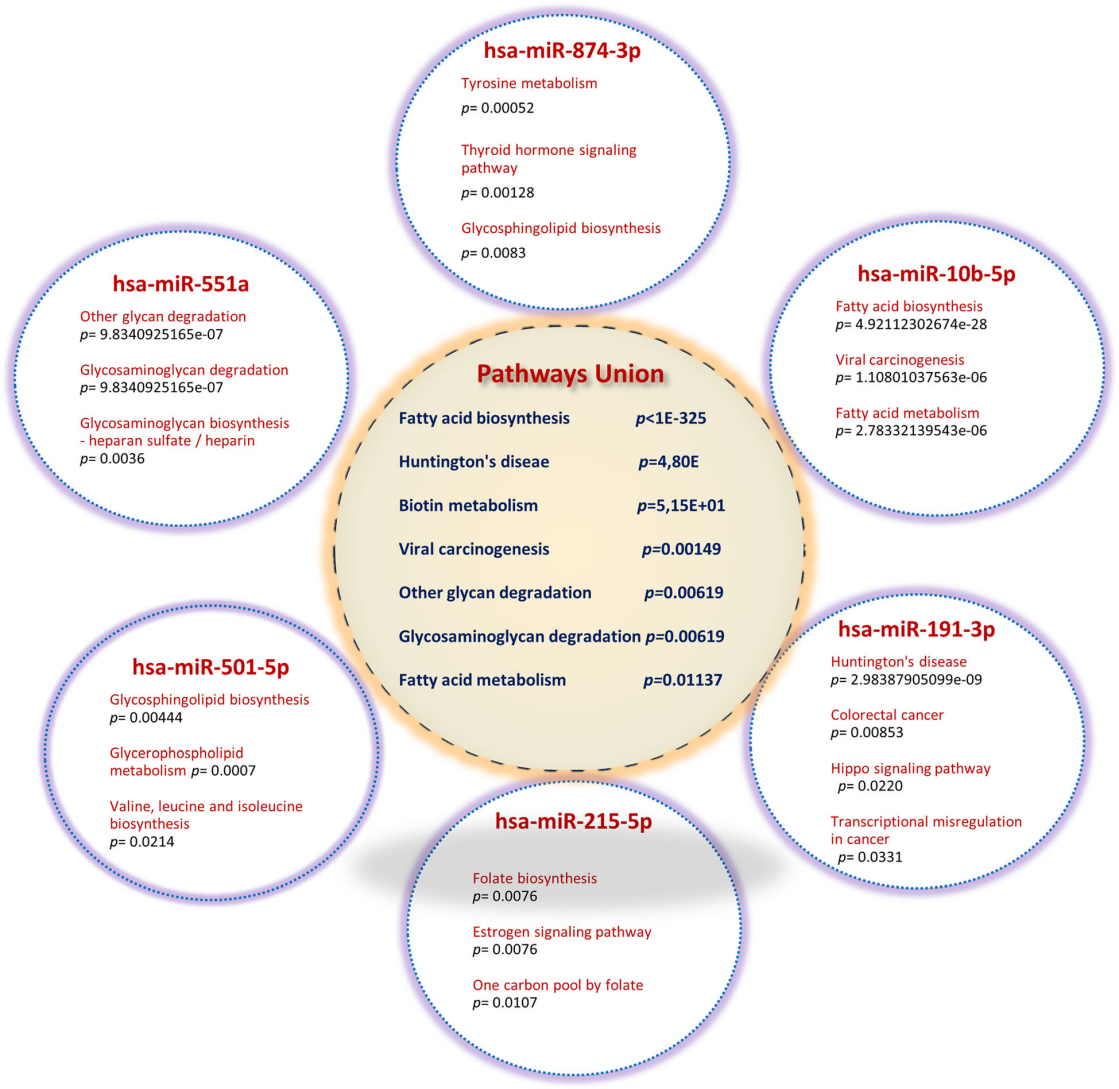
KEGG pathways of differentially expressed miRNAs between NW and OW/Ob. Pathways enrichment analysis of single mRNAs deregulated in compared groups. Pathways were classified according to KEGG functional annotations to identify top pathways that were actively regulated by miRNAs. Pathways union of six active miRNAs is also reported. The merged *p* value is extracted by combining calculated significance levels using Fisher’s exact test (hypergeometric distribution) with a *p* value threshold = 0.05 and microT threshold = 0.8

Target prediction and pathway enrichment was achieved using the software DIANA-miRPath v3.0. In detail, data analysis for multiple miRNAs was performed by the genes union selection. A *p* value threshold of 0.05 and FDR correction were applied to the analysis. miRNA target genes were processed by miRPath to find enriched biological pathways provided by KEGG. Moreover, target genes were classified according to KEGG functional annotations to identify top pathways that were actively regulated by miRNAs. All identified pathways were arranged according to enrichment statistical scores (*p* values) in addition to the number and names of miRNA target genes implicated in each KEGG pathway. Since both donor and target organs of circulating miRNAs are unknown, the top predicted pathways were explored either for single miRNAs or in their association (Fig. 5). Computational predictions of target genes followed by Gene Ontology (GO) enrichment analysis designated a pleiotropic role of these miRNAs in controlling relevant biological processes also including gene expression and cellular biosynthetic process (Additional file 2: Figure S2).

## Discussion

Numerous studies reveal a connection between epigenetic marks and human obesity. There is growing evidence that the epigenetic regulation of the gene expression represents a main contributor to the variation of predisposition to obesity and obesity-related disease [11]. As an example, epigenome-wide association studies have shown that BMI is connected with extensive changes in DNA methylation status and alteration in DNA methylation was confirmed to predict future development of T2D [12]. Other studies suggest that some of the methylation marks are a result of an obese phenotype and not necessarily the cause [13]. Nevertheless, evidence of the role of epigenetics in obesity comes mainly from animal models and studies with humans are still limited, but the results so far have shown promise to help explain the variation in predisposition to obesity. Although numerous aspects remain to be clarified, miRNAs research has contributed to shed lights on epigenetic mechanisms related to obesity. Circulating miRNAs are increasingly explored as innovative and non-invasive diagnostic markers. Of the 78,730 manuscripts regarding miRNAs to date annotated in PubMed, the majority has a straight association to human diseases and most to cancer. Several studies have clearly established that miRNAs correlate in a causative manner with obesity by directly affecting the status and functions of adipose tissue, pancreas, liver, muscle, and additional tissues and organs [14]. Remarkably, the screening of the tissue expression pattern of the five dysregulated miRNAs established that these miRNAs are amply co-expressed in plasma samples and other tissues (Additional file 3: Figure S3).

Additionally, a number of reports have assessed differences in circulating miRNAs content in overweight/obese and T2D individuals [1, 10, 15–18], and levels of definite miRNAs vary under different conditions [16, 19–25]. Interestingly, adipose tissue macrophages in Ob mice have been shown to secrete miRNA-containing exosomes which cause glucose intolerance and insulin resistance when administered to lean mice [26]. Moreover, connections between circulating miRNAs and obesity have been investigated in adult as well as in children by several studies [1, 7–9, 18, 27–29]. Within these investigations, characterized miRNAs have not been constantly confirmed and a number of inconsistencies have been reported among different studies, with discrepancies partially explained by dissimilarities in miRNAs detection procedures, experimental workflows, and cohorts selection criteria.

In the present study, as efficiency-optimized strategy, we performed a pre-screening in which compared groups were investigated for diverse countries as distinct pools. Of note, when miRNA signatures were analyzed by hierarchical clustering, a high degree of variability was detected among the different countries. As an example, the miRNAs identified as differentially expressed in OW/Ob children in a small sub-sample of the I.Family Italian cohort [19] were not confirmed in the whole European cohort. This result is not inconsistent with our overall hypotheses, since recent reports supporting the assumptions that levels of circulating miRNAs are likewise affected by lifestyle factors [30] and that many miRNA families are connected to diet and nutritional interventions [31]. Furthermore, it has been suggested that dietary miRNAs may also resist digestion and can be recognized in biofluids [32], even if the significance of food-derived miRNAs and their ability to affect cross-species miRNAs remains still questioned [33, 34]. Taken all together, these possibilities may speculatively contribute to explain the variability in circulating miRNA patterns from different countries.

During the last years, numerous efforts have been made to identify reliable and predictive non-invasive biomarkers to identify primary signs of metabolic disorders. Accordingly, to evaluate miRNAs performance in discriminating OW/Ob children, ROC curves were constructed for miRNAs. Single miRNAs, among experimentally confirmed, generally exhibited low AUC values with the highest values for miR-874-3p and miR-501-5p which used in combination provided an interesting value (AUC = 0.782).

Pearson’s analysis confirmed that, among the miRNAs identified in ANCOVA analysis, miR-10b-5p, miR-215-5p, miR-501-5p, miR-551a, and miR-874-3p significantly correlated with BMI *z*-score. No significant association between investigated biochemical parameters and differential expressed miRNAs was identified, with the exception of miR-191-3p which in Pearson’s analysis correlates with insulin levels and HOMA index. This result is in line with a previous research demonstrating its abnormal levels in both serum and plasma in T2D patients compared to healthy controls [16]. However, glucose homeostasis is a complex trait affected by multiple miRNAs which can change depending on which metabolically active tissue is affected.

In the current research, molecular interactions and functions of confirmed miRNAs were predicted using the miRPath analysis. Among the top predicted pathways, interaction with the target transcripts were further screened through the use of KEGG. This hypothesis-generating exercise suggests the involvement of characterized miRNAs in metabolic relevant pathways. Interestingly, the enzyme fatty acid synthase was the top predicted target of hsa-miR-10b-5p in the “fatty acid biosynthesis” (KEGG: hsa00061) and “fatty acid metabolism” (KEGG: hsa01212) pathways. Of note, this miRNA has also been shown to be differentially expressed during 3T3-L1 pre-adipocyte differentiation and may play active roles during adipogenesis [35]. Remarkably, a study in an animal model showed that miR-10b-5p represents the most abundant miRNA found in the subcutaneous adipose tissue [36].

Earlier studies confirmed the abnormal levels of miR-191-3p in T2D as reported above [16]. Moreover, it has been shown that miR-191 is considerably downregulated in adult peripheral T*reg* cells of diabetic patients compared with healthy individuals. Comprehensively, miR-191 represents an emerging player in disease biology since it regulates important cellular processes such as cell proliferation, differentiation, apoptosis by targeting relevant transcription factors, chromatin remodelers, and cell cycle-associated genes [37].

Among the top differentially expressed miRNAs, mir-874 has been recently connected to weight loss [38]. Interestingly, the thyroid hormone receptor alpha, the estrogen receptor 1, and the retinoid X receptor alpha (implicated in the adipocyte relevant PPAR signaling) were remarkable molecular target of hsa-miR-874-3p in the predicted “thyroid hormone signalling” (KEGG: hsa04919) pathway. Of note, the RXRA/PPARA heterodimer is required for PPARA transcriptional activity on fatty acid oxidation genes such as *ACOX1* and the cytochrome P450 system genes. Additionally, it has been shown that miR-874 is transcriptionally controlled by Foxo3a. Members of the FoxO family have been originally implicated in insulin/insulin-like growth factor signaling with effects of FoxOs on gene expression usually in the opposite direction as insulin and IGF-1. Of note, the catechol-O-methyltransferase and the dopa decarboxylase were among the top predicted target of hsa-miR-874-3p in the “tyrosine metabolism” (KEGG: hsa00350) pathway. Interestingly, changes of tyrosine over time were recently associated with metabolomic changes in childhood obesity; fascinatingly, tyrosine was identified as the most relevant metabolite in a random forest analysis in Ob children [39]. It is noteworthy that Src homology 2 B adaptor protein 1 (SH2B1) was a top predicted target of hsa-miR-874-3p. SH2B1 is a member of adaptor proteins influencing a variety of signaling pathways mediated by JAK and receptor tyrosine kinases. SH2B1 acts by performing classical adaptor functions recruiting specific proteins to activated receptors. Deletion of the SH2B1 gene in mice has been shown to result in a severe leptin resistance, obesity, insulin resistance, and T2D, demonstrating its critical role for the maintenance of normal body weight, insulin sensitivity, and glucose metabolism.

In this study, a deregulation of miR-215-5p was also established. Recent studies in 3T3-L1 cell-line demonstrated that miR-215-5p acts as a negative regulator of adipocyte differentiation through a post-transcriptional regulation of FNDC3B and CTNNBIP1 during early adipogenesis [40].

A further attractive speculation concerns hsa-miR-501-5p. The enzyme branched-chain amino acid transaminase (BCAA) 1A is among top predicted target of this miRNA in the “valine, leucine and isoleucine biosynthesis” (KEGG: hsa00290) pathway. Blood levels of the BCAAs are typically elevated in Ob, insulin-resistant humans, and models of diet-induced diabetes. Diet specifically reduced in BCAAs is sufficient to improve glucose tolerance and body composition, supporting the concept that BCAAs contribute to obesity and diabetes [41].

Mammalian glycans have been confirmed to be involved in molecular and cellular mechanisms governing health and disease, and changes in glycosylation have been observed in both genetic and acquired disease state [42]. In recent times, it has been proposed that flux through the hexosamine biosynthetic pathway may affect the development of insulin resistance and complications associated with diabetes, primarily through O-GlcNAcylation [43]. Noteworthy, the enzyme hexosaminidase was the top predicted target of hsa-miR-551a in the “glycan degradation” (KEGG: hsa00511) and “glycosaminoglycan biosynthesis” (KEGG: hsa00534) pathways.

Gene Ontology enrichment analysis designated selected miRNAs as key regulators of relevant biological processes. But, plasma levels do not necessarily reflect the effects exerted inside cells and tissues. Nevertheless, the concept that exosomal miRNAs can be transferred to other cell types and act through mechanisms of paracrine or endocrine regulation is supported by experimental evidence. Current experimental design cannot answer this question.

## Conclusions

The exciting emergence of circulating miRNAs as stable and accessible molecules opened a promising research avenue for the detection of non-invasive biomarkers. In line with this, our cross-sectional analysis showed that a panel of miRNAs is differentially expressed in overweight/low-grade obesity children and adolescents. The addition of covariates (age, sex, and country of origin) to linear regression models did not meaningfully attenuate the association for BMI *z*-score. Bioinformatics confirmed the possible role of recognized miRNAs to act as key regulators of metabolism, playing pivotal roles in early stages of obesity by directly affecting the status and functions of multiple candidate genes. However, evidence concerning how these molecules may act remains questioned, due to their ability to simultaneously regulate various pathways/gene networks, technical limitations of in vivo profiling, and the detected high degree of variability in miRNA levels, almost certainly due to individual lifestyle factors.

The planned follow-up of the I.Family cohort in prospectively designed studies will contribute to establish if selected miRNAs, measured at baseline, are valuable biomarkers for the early detection of subjects at risk of excess body fat accumulation with potential applications for a prompt diagnosis and grading of childhood obesity and related metabolic abnormalities.

## Methods

### Study population

The I.Family project (www.ifamilystudy.eu), aimed to assess the determinants of eating behavior in children and adolescents of eight European countries (Belgium, Cyprus, Estonia, Germany, Hungary, Italy, Spain, and Sweden) and related health outcomes, was built on the IDEFICS cohort (www.ideficsstudy.eu) established in 2007 and followed-up in 2013–2014. A full description of the project has been recently published [2]. This study was conducted according to the standards of the Declaration of Helsinki. Approval by the appropriate ethics committees was obtained by each of the eight participating centers carrying out the fieldwork. Participants were not subjected to any study procedure before both the children and their parents gave their oral (children) and written (parents) informed consent for examinations, collection of samples, subsequent analysis, and storage of personal data and collected samples. The study registration number is ISRCTN62310987.

Clinical data were collected from local participating centers using standardized procedures. A detailed description of the anthropometric measurements, including intra- and inter-observer reliability, has been previously published [44]. Weight was determined to the nearest 0.1 kg using an electronic scale (Tanita BC 420 SMA, Tanita Europe GmbH, Sindelfingen, Germany) with children wearing only light clothes without shoes. Height was measured using a calibrated stadiometer instrument (Seca 225, Seca GmbH&Co.KG., Hamburg, Germany) with an approximation of 0.1 cm. BMI was calculated as weight (in kg) divided by height squared (in m^2^). Sex and age-specific *z*-score BMI was calculated for each child and used for the statistical analysis. Children were classified as normal weight, overweight, or obese according to the cutoffs released by IOTF [45]. For the present study, in each country, we selected 20 children who retained normal weight, i.e., who showed a BMI *z*-score between − 1 and + 1 at baseline and follow-up and did not change more than ±0.1 in BMI *z*-score per year (defined as normal weight, NW), and 20 children who retained overweight or obesity, i.e., who had a BMI *z*-score of more than + 1 at baseline and follow-up, respectively, and did not change more than ± 0.1 in BMI *z*-score per year (defined as overweight/obese, OW/Ob).

### Sample processing and metabolic parameters

The fasting venous blood was collected in BD Vacutainer® blood collection tubes according to standard operating procedures. A detailed description of sample collection and analytical procedures has been published by Peplies et al. [46].

Total cholesterol, high-density lipoprotein cholesterol (HDL-C), triglyceride (TRG), glucose, glycated hemoglobin (HbA1c), and serum insulin levels were measured as part of routine laboratory testing, in a central laboratory (Laboratoriumsmedizin Dortmund Dr. Eberhard und Partner GbR). LDL cholesterol was calculated according to the formula of Friedwald. Insulin resistance was estimated by the homeostatic model assessment (HOMA) index calculated according to the following formula: HOMA = serum insulin (mU/l) × blood glucose (mg/dl)/405.

### Study design, miRNAs extraction, and profiling

Prior to miRNA extraction, spectrophotometry was carried out on plasma samples to test for hemolysis by measuring the absorbance of free hemoglobin at 414 nm; samples with OD414 greater than 0.2 were excluded from the study [29].

For miRNAs extraction, plasma samples were centrifuged at 1900×*g* for 10 min at 4 °C in a benchtop centrifuge (Eppendorf, Germany), aliquoted into 1.5 mL Eppendorf tubes and quickly stored at − 80 °C in the absence of freeze-thaw cycles until processing. Circulating miRNAs were evaluated in a three-step procedure: (A) a pooled miRNome determination, in which samples from NW and OW/Ob, grouped by countries, was analyzed as pools in triplicate by PCR arrays for miRNA profiling experiments; (B) an individual sample validation, in which the most relevant miRNAs in terms of fold changes and significance, was subsequently confirmed in RT-qPCR as distinct assays performed in triplicate; (C) a functional prediction, in which the molecular functions of differentially expressed miRNAs, was predicted by bioinformatics tools.

Circulating miRNAs were first isolated from plasma pooled samples, where the single country corresponds to a distinct pool, belonging to NW and OW/Ob groups, respectively. The miRNeasy Serum/Plasma Kit (Qiagen, Germany) was used according to the manufacturer’s instructions. To assess recoveries after RNA isolation, *Caenorhabditis elegans* miR-39 (Cel-miR-39) was added to each sample (5.6 × 10^8^ molecules) before the extraction process as *spike*-*in* control. cDNAs were generated by the dedicated miScript RTII kit (Qiagen, Germany) in the presence of the HiSpec Buffer (Qiagen, Germany) with miRNA specific stem-looped RT primers according to the manufacturer’s guidelines. All samples were extracted and processed in triplicate. cDNAs were investigated by the Human Serum & Plasma 384HC miRNA PCR Arrays (Qiagen, Germany) to assess 372 miRNAs typically detectable in serum and plasma following the manufacturer’s recommendations (Qiagen, Germany) by using a ViiA7 Real-Time PCR System (Applied Biosystems, Thermo Fisher Scientific, Waltham, MA, USA). Reaction conditions were as follows: 15 min at 95 °C and 40 cycles of 15 s at 94 °C, 30 s at 60 °C, and 30 s at 72 °C. All assays were inspected for distinct melting curves, and the Tm was checked. The reverse transcription and amplification efficiencies were also calculated to verify the absence of interfering compounds. *C*_t_ values > 35 were considered as negative amplification. In addition, level of miR-451a, highly abundant in RBCs, was preliminarily assessed by qPCR. Both miR-451a and miR-23a-3p were measured in all plasma samples. Samples with a ΔCq ratio of ≥ 7 were excluded from our analysis [47].

Arrays were analyzed by the Web-based miScript Arrays data analysis software package (SABiosciences, Qiagen, https://www.qiagen.com/ch/shop/genes-and-pathways/data-analysis-center-overview-page/). References for data normalization included the spike-in Cel-miR-39 in addition to SNORD95 that has been verified to hold a relatively stable expression. Normalization of expression was done using the geometric mean of the controls.

A set of candidate deregulated miRNAs was selected and further validated by individual assays using the miScript Primer Assays in combination with the miScript PCR kit according to the manufacturer’s recommendations (Qiagen, Germany). Confirmatory real-time quantitative RT-PCR (RT-qPCR) was carried out using the SYBR green technology; samples were analyzed in triplicate using the following conditions: 95 °C for 15 min, followed by 40 cycles of 94 °C for 15 s, 55 °C for 30 s, and 70 °C for 30 s. All *C*_t_ values above 38 were set to 38 as the maximum value and regarded as non-amplification. miRNA levels were determined using the Cel-miR-39 as the endogenous normalizer. Relative levels were calculated using Data Assist v3.1 software package (Life Technologies, Thermo Fisher Scientific, Italy).

### Bioinformatics

Obesity-associated miRNAs were explored using the target prediction tool miRPath v3.0. MiRPath pipeline achieves advanced analysis such as hierarchical clustering of miRNAs and pathways based on the levels of their interactions. miRNA targets (in CDS or 3′-UTR regions) included predictions from DIANA algorithm (microT-CDS) and also experimentally validated miRNA interactions derived from DIANA-TarBase v7.0. Predicted or validated interactions were subsequently combined by merging and by using meta-analysis algorithms [48]. All predicted targets were further analyzed through the use of the Kyoto Encyclopedia of Genes and Genomes (KEGG) which embraces a large database of biological and chemical relationships extracted from scientific literature. Interaction networks were further validated by miRTargetLink Human.

The distribution of the miRNAs in human tissues was assessed using the MiRmine Human miRNA Expression Database (http://guanlab.ccmb.med.umich.edu/mirmine/index.html).

### Statistical analysis

The first step of the analysis was performed on the pooled samples belonging to NW and OW/Ob groups, respectively. The Benjamini-Hochberg false discovery rate method was adopted in Web-based miScript Arrays data analysis software package for multiple comparisons to check whether any differences in the levels of miRNAs between compared groups were statistically significant.

The second step was the validation on the individual samples of miRNAs identified on the pooled samples. Statistical analyses on the selected candidates miRNAs were performed by using IBM SPSS Statistics (v23.0.; IBM Corp). Data were calculated as means and standard deviation (SD) or means and 95% confidence intervals (CI), as indicated. A two-tailed *p* value less than 0.05 was considered statistically significant. Biochemical differences between compared groups were determined using analysis of variance (one-way ANOVA). Analysis of miRNAs expression was performed using analysis of covariance (ANCOVA) adjusting for covariates (age, sex, and country of origin).

Pearson’s analysis was used to study associations between the variables. For selected miRNAs, a receiver–operator curve (ROC) was constructed and area under the curve (AUC) was calculated to evaluate the sensitivity and specificity for predicting cases (Multibase v2015, Numerical Dynamics).

## Additional files

Additional file 1: **Figure S1.** Candidate miRNAs selection. A) Scatter plot analysis. The scatter plot analysis compares the normalized expression of each miRNA present on the array between the selected groups (OW/Ob vs NW) by plotting them against one another to visualize changes in miRNA levels. The central line indicates unchanged expression. The boundary (fold-change cut-off) was set to 1.7. The red circles are over-expressed miRNAs and the green circles are under-expressed miRNAs. Several miRNAs were annotated. B) Volcano plot analysis. For each circulating miRNAs, significance is indicated by negative log_10_ *p*-value on the y-axis, and the standardized difference in log_2_ C_t_ scores on x-axis. The fold-change cut-off was set to 1.7 and *p*-value to 0.05. Several miRNAs were annotated. The red circles are over-expressed miRNAs in Ow/Ob and the green circles are under-expressed miRNAs. (TIF 504 kb)

Additional file 2: **Figure S2.** Gene Ontology categories. Target genes were mapped to the Gene Ontology categories to gain a high-level view of gene functions possibly affected by the altered miRNAs expression. The color-key at the top represents the log *p*-values. (TIF 1457 kb)

Additional file 3: **Figure S3.** Expression of miRNAs in different Human Tissues. (JPEG 430 kb)

## Abbreviations

AUC: Area under the curve
BMI: Body mass index
Cel-miR-39: *Caenorhabditis elegans* miR-39
FDR: False discovery rate
GO: Gene Ontology
HbA1c: Glycated hemoglobin
HDL: High-density lipoprotein
HOMA: Homeostatic model assessment
KEGG: Kyoto Encyclopedia of Genes and Genomes
LDL: Low-density lipoprotein
miRNA: microRNA
NW: Normal weight
OW/Ob: Overweight/obese
ROC: Receiver–operator curve
RT-qPCR: Real-time quantitative RT-PCR
T2D: Type 2 diabetes
TG: Triglycerides

## References

[1] Iacomino G, Russo P, Stillitano I, Lauria F, Marena P, Ahrens W, De Luca P, Siani A (2016). Circulating microRNAs are deregulated in overweight/obese children: preliminary results of the I.Family study. Genes Nutr.

[2] Ahrens W, Siani A, Adan R, De Henauw S, Eiben G, Gwozdz W, Hebestreit A, Hunsberger M, Kaprio J, Krogh V (2017). Cohort profile: The transition from childhood to adolescence in European children-how I.Family extends the IDEFICS cohort. Int J Epidemiol.

[3] Hausser J, Zavolan M (2014). Identification and consequences of miRNA-target interactions--beyond repression of gene expression. Nat Rev Genet.

[4] Gurtan AM, Sharp PA (2013). The role of miRNAs in regulating gene expression networks. J Mol Biol.

[5] Turchinovich A, Weiz L, Langheinz A, Burwinkel B (2011). Characterization of extracellular circulating microRNA. Nucleic Acids Res.

[6] Kim YK (2015). Extracellular microRNAs as biomarkers in human disease. Chonnam Med J.

[7] Can U, Buyukinan M, Yerlikaya FH (2016). The investigation of circulating microRNAs associated with lipid metabolism in childhood obesity. Pediatr Obes..

[8] Prats-Puig A, Ortega FJ, Mercader JM, Moreno-Navarrete JM, Moreno M, Bonet N, Ricart W, Lopez-Bermejo A, Fernandez-Real JM (2013). Changes in circulating microRNAs are associated with childhood obesity. J Clin Endocrinol Metab.

[9] Thompson MD, Cismowski MJ, Serpico M, Pusateri A, Brigstock DR. Elevation of circulating microRNA levels in obese children compared to healthy controls. Clin Obes. 2017. 10.1111/cob.12192. 10.1111/cob.12192 28397375

[10] Iacomino G, Siani A (2017). Role of microRNAs in obesity and obesity-related diseases. Genes Nutr.

[11] van Dijk SJ, Molloy PL, Varinli H, Morrison JL, Muhlhausler BS (2015). Epigenetics and human obesity. Int J Obes.

[12] van Dijk SJ, Tellam RL, Morrison JL, Muhlhausler BS, Molloy PL (2015). Recent developments on the role of epigenetics in obesity and metabolic disease. Clin Epigenetics.

[13] Wahl S, Drong A, Lehne B, Loh M, Scott WR, Kunze S, Tsai PC, Ried JS, Zhang W, Yang Y (2017). Epigenome-wide association study of body mass index, and the adverse outcomes of adiposity. Nature.

[14] Kunej T, Jevsinek Skok D, Zorc M, Ogrinc A, Michal JJ, Kovac M, Jiang Z (2013). Obesity gene atlas in mammals. J Genomics.

[15] Ortega FJ, Mercader JM, Catalan V, Moreno-Navarrete JM, Pueyo N, Sabater M, Gomez-Ambrosi J, Anglada R, Fernandez-Formoso JA, Ricart W (2013). Targeting the circulating microRNA signature of obesity. Clin Chem.

[16] Zampetaki A, Kiechl S, Drozdov I, Willeit P, Mayr U, Prokopi M, Mayr A, Weger S, Oberhollenzer F, Bonora E (2010). Plasma microRNA profiling reveals loss of endothelial miR-126 and other microRNAs in type 2 diabetes. Circ Res.

[17] Pescador N, Perez-Barba M, Ibarra JM, Corbaton A, Martinez-Larrad MT, Serrano-Rios M (2013). Serum circulating microRNA profiling for identification of potential type 2 diabetes and obesity biomarkers. PLoS One.

[18] Cui X, You L, Zhu L, Wang X, Zhou Y, Li Y, Wen J, Xia Y, Wang X, Ji C, et al. Change in circulating microRNA profile of obese children indicates future risk of adult diabetes. Metabolism. 2017. 10.1016/j.metabol.2017.09.006. 10.1016/j.metabol.2017.09.006 28966078

[19] Guay C, Regazzi R (2013). Circulating microRNAs as novel biomarkers for diabetes mellitus. Nat Rev Endocrinol.

[20] Wang YT, Tsai PC, Liao YC, Hsu CY, Juo SH (2013). Circulating microRNAs have a sex-specific association with metabolic syndrome. J Biomed Sci.

[21] Zile MR, Mehurg SM, Arroyo JE, Stroud RE, DeSantis SM, Spinale FG (2011). Relationship between the temporal profile of plasma microRNA and left ventricular remodeling in patients after myocardial infarction. Circ Cardiovasc Genet.

[22] Cermelli S, Ruggieri A, Marrero JA, Ioannou GN, Beretta L (2011). Circulating microRNAs in patients with chronic hepatitis C and non-alcoholic fatty liver disease. PLoS One.

[23] Li S, Zhu J, Zhang W, Chen Y, Zhang K, Popescu LM, Ma X, Lau WB, Rong R, Yu X (2011). Signature microRNA expression profile of essential hypertension and its novel link to human cytomegalovirus infection. Circulation.

[24] Karolina DS, Tavintharan S, Armugam A, Sepramaniam S, Pek SL, Wong MT, Lim SC, Sum CF, Jeyaseelan K (2012). Circulating miRNA profiles in patients with metabolic syndrome. J Clin Endocrinol Metab.

[25] Deiuliis JA (2016). MicroRNAs as regulators of metabolic disease: pathophysiologic significance and emerging role as biomarkers and therapeutics. Int J Obes.

[26] Ying W, Riopel M, Bandyopadhyay G, Dong Y, Birmingham A, Seo JB, Ofrecio JM, Wollam J, Hernandez-Carretero A, Fu W (2017). Adipose tissue macrophage-derived exosomal miRNAs can modulate in vivo and in vitro insulin sensitivity. Cell.

[27] Heneghan HM, Miller N, McAnena OJ, O'Brien T, Kerin MJ (2011). Differential miRNA expression in omental adipose tissue and in the circulation of obese patients identifies novel metabolic biomarkers. J Clin Endocrinol Metab.

[28] Carreras-Badosa G, Bonmati A, Ortega FJ, Mercader JM, Guindo-Martinez M, Torrents D, Prats-Puig A, Martinez-Calcerrada JM, Platero-Gutierrez E, De Zegher F (2015). Altered circulating miRNA expression profile in pregestational and gestational obesity. J Clin Endocrinol Metab.

[29] Ouyang S, Tang R, Liu Z, Ma F, Li Y, Wu J (2017). Characterization and predicted role of microRNA expression profiles associated with early childhood obesity. Mol Med Rep.

[30] Slattery ML, Herrick JS, Mullany LE, Stevens JR, Wolff RK (2017). Diet and lifestyle factors associated with miRNA expression in colorectal tissue. Pharmgenomics Pers Med.

[31] Palmer JD, Soule BP, Simone BA, Zaorsky NG, Jin L, Simone NL (2014). MicroRNA expression altered by diet: can food be medicinal?. Ageing Res Rev.

[32] Liang G, Zhu Y, Sun B, Shao Y, Jing A, Wang J, Xiao Z (2014). Assessing the survival of exogenous plant microRNA in mice. Food Sci Nutr.

[33] Pastrello C, Tsay M, McQuaid R, Abovsky M, Pasini E, Shirdel E, Angeli M, Tokar T, Jamnik J, Kotlyar M (2016). Circulating plant miRNAs can regulate human gene expression in vitro. Sci Rep.

[34] Witwer KW, Zhang CY (2017). Diet-derived microRNAs: unicorn or silver bullet?. Genes Nutr.

[35] Kajimoto K, Naraba H, Iwai N (2006). MicroRNA and 3T3-L1 pre-adipocyte differentiation. RNA.

[36] Mentzel CM, Anthon C, Jacobsen MJ, Karlskov-Mortensen P, Bruun CS, Jorgensen CB, Gorodkin J, Cirera S, Fredholm M (2015). Gender and obesity specific microRNA expression in adipose tissue from lean and obese pigs. PLoS One.

[37] Nagpal N, Kulshreshtha R (2014). miR-191: an emerging player in disease biology. Front Genet.

[38] Milagro FI, Miranda J, Portillo MP, Fernandez-Quintela A, Campion J, Martinez JA (2013). High-throughput sequencing of microRNAs in peripheral blood mononuclear cells: identification of potential weight loss biomarkers. PLoS One.

[39] Butte NF, Liu Y, Zakeri IF, Mohney RP, Mehta N, Voruganti VS, Goring H, Cole SA, Comuzzie AG (2015). Global metabolomic profiling targeting childhood obesity in the Hispanic population. Am J Clin Nutr.

[40] Peng Y, Li H, Li X, Yu S, Xiang H, Peng J, Jiang S (2016). MicroRNA-215 impairs adipocyte differentiation and co-represses FNDC3B and CTNNBIP1. Int J Biochem Cell Biol.

[41] Fontana L, Cummings NE, Arriola Apelo SI, Neuman JC, Kasza I, Schmidt BA, Cava E, Spelta F, Tosti V, Syed FA (2016). Decreased consumption of branched-chain amino acids improves metabolic health. Cell Rep.

[42] Dennis JW, Brewer CF (2013). Density-dependent lectin-glycan interactions as a paradigm for conditional regulation by posttranslational modifications. Mol Cell Proteomics.

[43] Ryczko MC, Pawling J, Chen R, Abdel Rahman AM, Yau K, Copeland JK, Zhang C, Surendra A, Guttman DS, Figeys D (2016). Metabolic reprogramming by hexosamine biosynthetic and Golgi N-glycan branching pathways. Sci Rep.

[44] Stomfai S, Ahrens W, Bammann K, Kovacs E, Marild S, Michels N, Moreno LA, Pohlabeln H, Siani A, Tornaritis M (2011). Intra- and inter-observer reliability in anthropometric measurements in children. Int J Obes.

[45] Cole TJ, Lobstein T (2012). Extended international (IOTF) body mass index cut-offs for thinness, overweight and obesity. Pediatr Obes.

[46] Peplies J, Fraterman A, Scott R, Russo P, Bammann K (2010). Quality management for the collection of biological samples in multicentre studies. Eur J Epidemiol.

[47] Blondal T, Jensby Nielsen S, Baker A, Andreasen D, Mouritzen P, Wrang Teilum M, Dahlsveen IK (2013). Assessing sample and miRNA profile quality in serum and plasma or other biofluids. Methods.

[48] Vlachos IS, Zagganas K, Paraskevopoulou MD, Georgakilas G, Karagkouni D, Vergoulis T, Dalamagas T, Hatzigeorgiou AG (2015). DIANA-miRPath v3.0: deciphering microRNA function with experimental support. Nucleic Acids Res.

