# Supplementary figures and images for: Circulating microRNAs are associated with early childhood obesity: results of the I.Family Study
Source: Genes Nutr. 2019 Jan 9;14:2. doi: 10.1186/s12263-018-0622-6 (PMC6327413; doi:10.1186/s12263-018-0622-6)

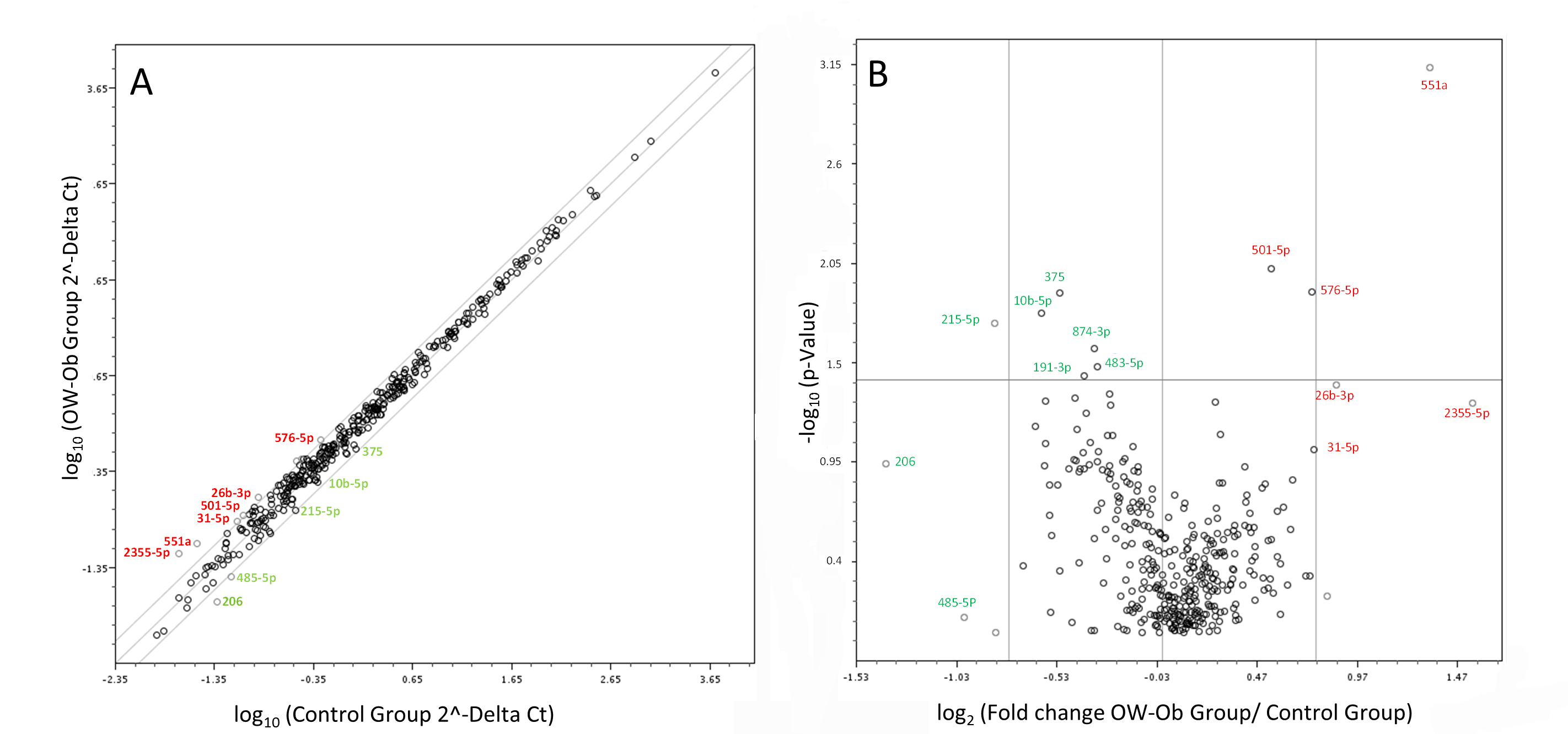

Supplement: Supplementary file 1 — Figure S1. Candidate miRNAs selection. A) Scatter plot analysis. The scatter plot analysis compares the normalized expression of each miRNA present on the array between the selected groups (OW/Ob vs NW) by plotting them against one another to visualize changes in miRNA levels. The central line indicates unchanged expression. The boundary (fold-change cut-off) was set to 1.7. The red circles are over-expressed miRNAs and the green circles are under-expressed miRNAs. Several miRNAs were annotated. B) Volcano plot analysis. For each circulating miRNAs, significance is indicated by negative log10 p-value on the y-axis, and the standardized difference in log2 Ct scores on x-axis. The fold-change cut-off was set to 1.7 and p-value to 0.05. Several miRNAs were annotated. The red circles are over-expressed miRNAs in Ow/Ob and the green circles are under-expressed miRNAs. (TIF 504 kb) [file 12263_2018_622_MOESM1_ESM.tif]

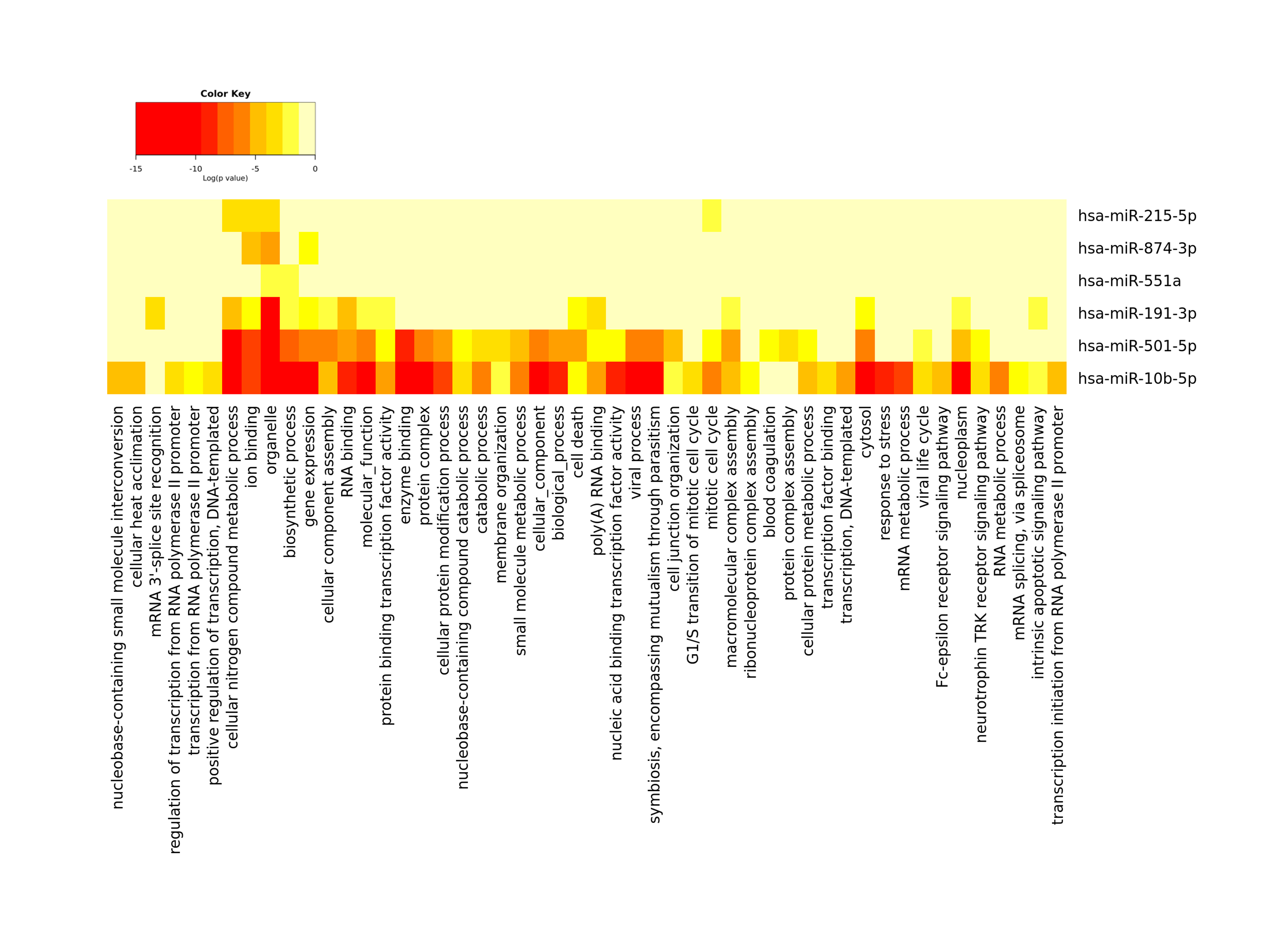

Supplement: Supplementary file 2 — Figure S2. Gene Ontology categories. Target genes were mapped to the Gene Ontology categories to gain a high-level view of gene functions possibly affected by the altered miRNAs expression. The color-key at the top represents the log p-values. (TIF 1457 kb) [file 12263_2018_622_MOESM2_ESM.tif]

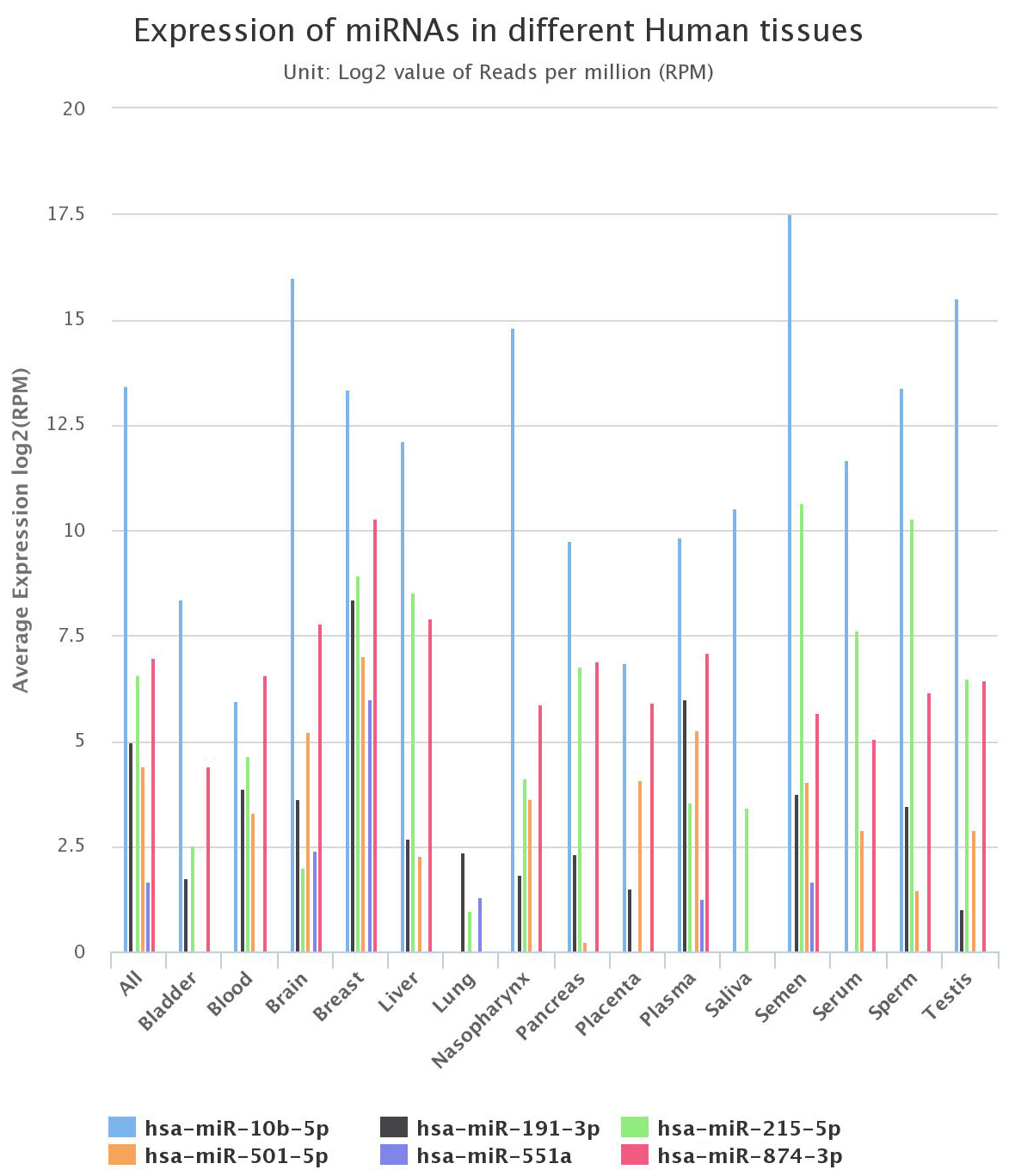

Supplement: Supplementary file 3 — Figure S3. Expression of miRNAs in different Human Tissues. (JPEG 430 kb) [file 12263_2018_622_MOESM3_ESM.jpeg]
